# Invariant errors reveal limitations in motor correction rather than constraints on error sensitivity

**DOI:** 10.1038/s42003-018-0021-y

**Published:** 2018-03-22

**Authors:** Hyosub E. Kim, J. Ryan Morehead, Darius E. Parvin, Reza Moazzezi, Richard B. Ivry

**Affiliations:** 1 Department of Psychology, University of California, Berkeley, Berkeley, CA 94720 USA; 2 Helen Wills Neuroscience Institute, University of California, Berkeley, Berkeley, CA 94720 USA; 3 John A. Paulson School of Engineering and Applied Sciences, Harvard University, Cambridge, MA 02138 USA; 4 Independent Investigator, Berkeley, CA 94720 USA

## Abstract

Implicit sensorimotor adaptation is traditionally described as a process of error reduction, whereby a fraction of the error is corrected for with each movement. Here, in our study of healthy human participants, we characterize two constraints on this learning process: the size of adaptive corrections is only related to error size when errors are smaller than 6°, and learning functions converge to a similar level of asymptotic learning over a wide range of error sizes. These findings are problematic for current models of sensorimotor adaptation, and point to a new theoretical perspective in which learning is constrained by the size of the error correction, rather than sensitivity to error.

## Abstract (web summary)

Kim *et al.* report a study of implicit sensorimotor adaptation using task-irrelevant clamped visual feedback and find that learning is constrained primarily by the size of the error correction rather than sensitivity to error. The results present a challenge to current models of adaptation and suggest a new framework to guide new models of this process.

## Introduction

Movement errors are ubiquitous, arising from numerous sources such as motor noise, fatigue, or changes in the environment. A large body of evidence has revealed that the motor system compensates for errors via sensorimotor adaptation^1^. This implicit learning process is thought to be driven by sensory prediction error, the discrepancy between the actual and predicted sensory outcome of a motor command^2–6^. A core issue for models of adaptation has centered on how this error signal is used to modify motor output^7–10^.

In classic models of sensorimotor adaptation, the response to error is assumed to be linear, with trial-by-trial corrections a constant fraction of error size^9,11^. The theoretical foundation for this relationship centers on the delta learning rule, where the weights between putative sensorimotor neurons are updated as a function of the magnitude of the difference between the actual and predicted output^9,12,13^. A standard formulation of this type of model is given by the following state-space equation:

$$z_{n + 1} = Az_n + {\it{Be}}_n$$

where *z*_*n*_ represents the state estimate of the perturbation on trial *n*, and *A* is a retention factor, the proportion of the state retained from one trial to the next. The error term, *e*, is multiplied by a scalar learning rate, *B*, to determine the change in the state estimate from trial-to-trial due to sensory prediction errors. However, empirical studies have shown limitations with the assumption of a constant learning rate. When operationalized as the ratio of the change in behavior relative to the error, sensitivity appears to be high for small errors, with the system correcting for a relatively large fraction of the error, and then rapidly decreases as errors become large^8,10,14,15^.

**Fig. 1:**
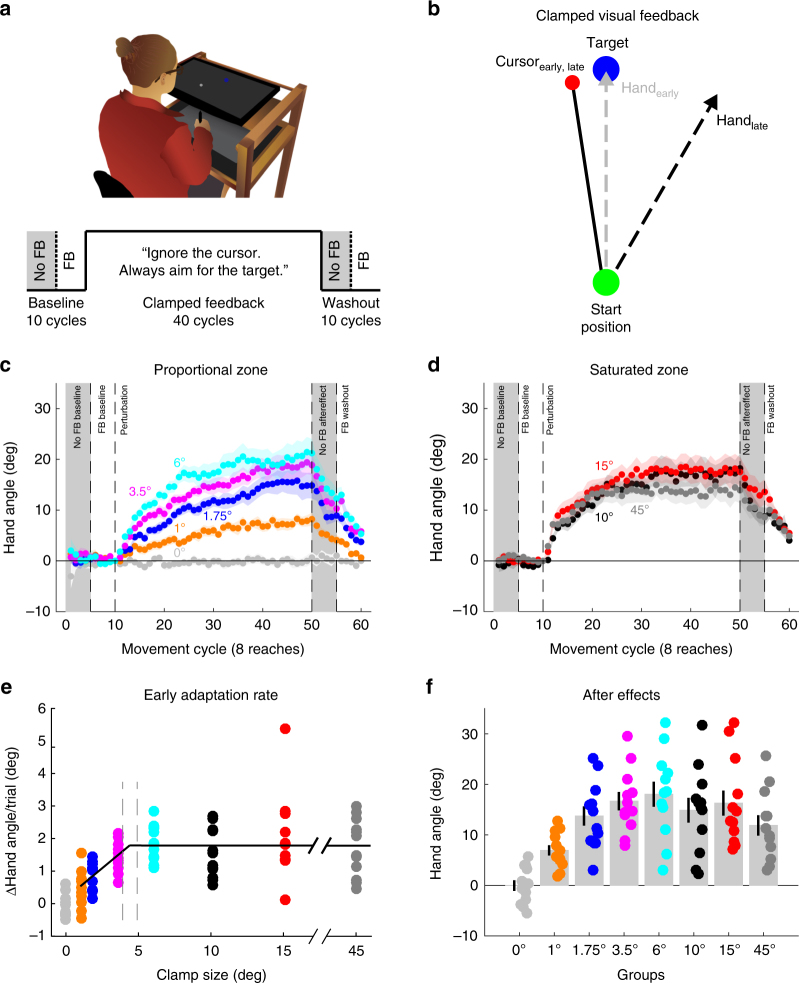
Initial adaptation rates scale with error size, yet saturate to an invariant response magnitude. **a** Illustration of experimental apparatus and task structure for Exp. 1. **b** Schematic view of clamped visual feedback paradigm, in which the angular path of the cursor is independent of hand movement direction. **c**, **d** Behavior for all groups (*n* = 12 per group), divided into two panels for visualization purposes. The small clamp groups (**c**) demonstrate adaptation rates which scale with error size, whereas the large clamp groups (**d**) show saturated responses. **e** Segmented regression indicates that the initial adaptation rate scales between 0° and 4.4° before saturating for all errors above this break point (dashed vertical lines represent 95% CI). **f** Sensorimotor aftereffects, measured during the first cycle following the clamp block. Dots are individuals; shading and error bars denote SEM. Gray shading denotes cycles without visual feedback

However, due to potential confounds in standard sensorimotor adaptation tasks, estimates of the error sensitivity function in many of these studies may be contaminated by other learning processes, such as the use of explicit aiming strategies^16^. To study adaptation without interference from explicit learning or performance-driven corrections, we recently introduced a method in which the visual feedback is task irrelevant and invariant over the course of the experiment^17^ (Fig. 1a, b). Despite full knowledge of the task-irrelevant clamped visual feedback, participants implicitly produce a marked change in performance. As shown in our initial study with this method, performance changes resulting from clamped feedback bear the classic hallmarks of adaptation, including sign-dependent corrections, persistent aftereffects, local generalization, and a dependency on the integrity of the cerebellum.

The use of clamped visual feedback offers a new tool to address a fundamental problem in error-based learning, namely, how the response of the system varies as a function of error size. In studies using a fixed, task-relevant perturbation (e.g., standard visuomotor rotation), error size is confounded with learning. As learning unfolds, the mean error size becomes dramatically smaller. To provide a cleaner assay of the responsiveness of the adaptation system to errors of varying size, previous studies have used errors that vary randomly in size and direction from trial to trial, such that the overall mean error is zero^10,14,15^. A limitation with this approach is that one can only measure trial-by-trial changes. In contrast, with the clamp method, we can examine the full accumulated adaptive response to errors of a given size since the error signal remains invariant. Thus, we can assess not only how changes in error magnitude influence the response of the system, but also how this responsiveness might change over time and training.

Our initial study with clamped visual feedback revealed learning functions that were surprisingly invariant over a wide range of error sizes (7.5–95°)^17^. This invariance was evident in the initial rate of adaptation as well as in the final asymptotic value. As noted above, prior studies indicate that sensitivity is reduced to large errors^8,10,14,15^; it may be that the smallest value previously tested with the clamp method (i.e., 7.5°) falls within the range in which the error-driven response is already saturated.

In the current study, we focus on small clamped errors (i.e., errors<7.5°), using perturbation sizes that are more representative of the feedback that we typically experience from intrinsic motor variability^18^. We expect that the response to these smaller clamped errors will be dependent on the size of the error, and thus, allow us to estimate the saturation point. Assuming we observe some scaling of the response as a function of error size, the clamp method also allows us to ask if this is evident in both the learning rate and asymptote as predicted by current models of adaptation.

## Results

### Initial adaptation rates only scale with error size for small errors

In a between-subject design, participants (*n* = 96, 12 per group) were presented with visual feedback that was clamped to a fixed path which was angularly offset from the target by 0°, 1°, 1.75°, 3.5°, 6°, 10°, 15°, or 45°. This manipulation was explicitly described to the participants and they were instructed to ignore the feedback and simply move directly to the target (Fig. 1a). With the exception of the 0° control group, all groups implicitly adapted to the clamp (*t*_11_ = −0.04, *p* = 0.97 for 0° group; *t*_11_ > 5.9, *p* < 0.0001 for all other groups; Fig. 1c, d).

Within all adapting groups, there was an effect of clamp size on the average per-trial rate of learning over the first five cycles (ANOVA: *F*_6,77_ = 6.45, *p* < 0.0001, *ƞ*^2^ = 0.33). Although there was a modest linear relationship between clamp size and early adaptation rate (*r*_82_ = 0.29, *p* = 0.01), the adaptation rate appeared to be composed of two zones, one where the rate scaled in proportion to error size, and another where rates were invariant. To formally assess this hypothesis, we performed segmented linear regressions. Taking model complexity into account, a two-region segmented regression yielded the best model (Supplementary Fig. 1). This model predicted that the break point between the proportional and saturated zones was at the remarkably low value of 4.4° (95% CI (3.9°, 4.9°), Fig. 1e).

These results, in combination with previous work^8,10,14,15,17^, are clearly at odds with models entailing a fixed learning rate (i.e., adaptation scaling linearly with error size). Prior observations of a nonlinear response to error have inspired models in which the learning rate saturates for large errors^13^, or large errors are discounted before the update step of the learning process^10,14,19^. If exposed to a constant perturbation, these models can generate similar adaptation rates and asymptotes in response to large errors. However, these models would also predict a lower asymptote in response to small errors in the linearly proportional zone. Contrary to this prediction, with the exception of the 1° group, the magnitude of adaptation at the end of training was similar for a wide range of clamp offsets (Fig. 1f and Supplementary Note 1).

### Adaptation converges on a common asymptote

**Fig. 2:**
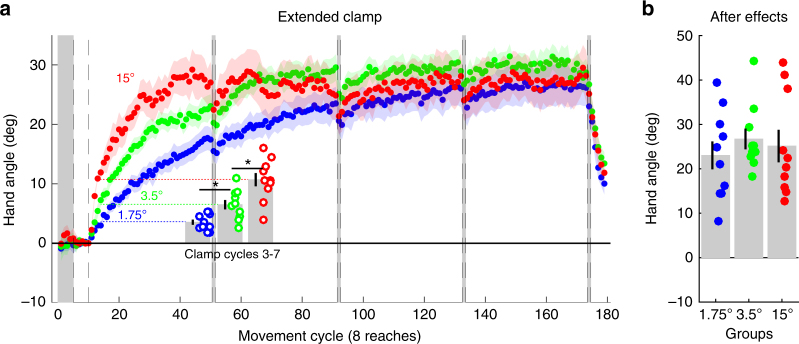
Implicit adaptation converges on a common asymptote. **a** The 1.75°, 3.5°, and 15° clamp groups in Exp. 2 (*n* = 10 per group) adapted at markedly different rates (bar graphs depict mean of cycles 3–7). However, there was convergence of all three learning functions by the end of 160 cycles, and (**b**) no difference between groups in the size of the final aftereffects. Asterisk in **a** denotes significant differences between groups early in the clamp phase. Dots are individuals; shading and error bars denote SEM

The observation of similar performance across a range of error sizes at the end of training is tempered by the fact that we did not have a sufficient number of trials to ensure that learning had become asymptotic; as such, it is unclear if prolonged exposure to constant errors of varying size will converge at a common asymptote. To address this issue, we conducted a second experiment in which the number of cycles was increased from 40 to 160. Participants (*n* = 30, 10 per group) were exposed to clamped feedback with an angular offset of 1.75°, 3.5°, or 15°. These offsets were chosen because they span the range of early adaptation rates observed in Experiment 1 (Methods section). Consistent with the results of Experiment 1, there was a clear scaling of the rates across the proportional zone (ANOVA: *F*_2,27_ = 18.6; *p* < 0.0001; *ƞ*^2^ = .58), with Tukey–Kramer post hoc tests revealing significant differences between all pairwise comparisons (Fig. 2a). Strikingly, the three groups reached a similar asymptote, with all groups demonstrating final aftereffects of ~25° (ANOVA: *F*_2,27_ = 0.39, *p* = 0.68; *ƞ*^2^ = 0.03; Fig. 2b; see also Supplementary Note 2).

## Discussion

This dissociation between size-dependent early adaptation rates and invariant asymptotic adaptation is at odds with models that correct for a constant fraction of error size as well as error discounting models (Supplementary Fig. 2 and Supplementary Note 3). Even if the learning rate varies as a function of error size, assuming a fixed retention factor, these models predict that asymptotic behavior will diverge since the asymptote is determined by the equilibrium between learning and forgetting.

**Fig. 3:**
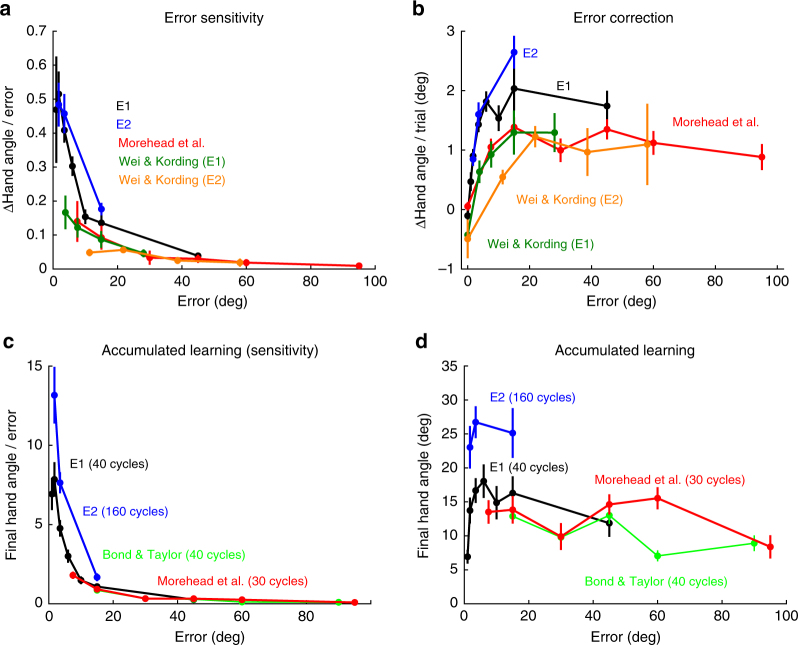
Adaptation assessed in terms of error sensitivity (left) or error correction (right). Here we plot data from several different studies^10,17,23^, including the present one, using two ways to consider trial-by-trial changes in hand angle as a function of error size (Methods section). **a** Error sensitivity, operationalized as the change in hand angle divided by error size, starts at an early maximum and quickly decays as errors increase in size. **b** The same data, plotted in terms of the untransformed error correction, shows a function that starts small and then saturates, suggesting that the motor system continues to produce a robust, invariant response over a wide range of error sizes. Plotting the aftereffect data in terms of a sensitivity function (**c**) also fails to capture the relative invariance of these data within a given experimental context (**d**). Note the one discrepant point from Exp. 1 in panels C and D from the 1° clamp condition; we suspect this is due to an insufficient number of trials to approximate asymptotic performance. Error bars denote SEM.

In addition to identifying a fundamental limitation with current models of sensorimotor adaptation, our results draw attention to a more general issue. Behavioral responses to error are usually interpreted through the lens of error sensitivity. This perspective is apparent not only in studies of visuomotor adaptation, but is also evident in research on saccadic^20^, locomotor^21^, and force field adaptation^22^. The sensitivity function is generated by dividing the magnitude of the motor correction by the error size. When applied to the behavioral data that we and others have observed, this divisive operation generates a function in which sensitivity is high for small errors and gradually decreases to near zero for large errors (Fig. 3a, c).

Although this error sensitivity metric is mathematically capable of approximating the behavioral effects observed with variation in error size, focusing on the untransformed behavioral responses to errors of varying size suggests a different perspective on the limiting factor in adaptation. As seen in Fig. 3b, adjustments in motor output scale for small errors before quickly reaching a saturation point that holds across a broad range of larger errors (Fig. 3b). Depicting the actual behavioral change from sensory prediction error highlights the limited dependency of the system on error size, as well as the common asymptotic level of learning in response to small and large errors (Fig. 3d). Thus, incorporating an update rule in which the correction (i.e., behavioral change), rather than error sensitivity, is modeled as a function of error size may offer a more appropriate framework for understanding the constraints underlying sensorimotor adaptation. The data in Fig. 3b suggest that the error correction function, expressed in terms of absolute change in heading direction from trial-to-trial, would have a half-sigmoid shape with a saturation point at a small error size.

We can envision three, non-mutually exclusive ways in which current models of adaptation could be modified to capture size-dependent early adaptation rates for small errors combined with invariant asymptotic adaptation. First, the learning rate and retention parameters could be coupled, scaling together with error size^24^. For instance, small errors may elicit smaller corrections and greater retention, while large errors may elicit larger corrections but weaker retention. Whereas current models have considered that learning rate may be dependent on error size, this variant would require that the forgetting process is also error size dependent (the *A* term in the state-space model equation). Moreover, to achieve a common asymptote across different error sizes constrains the form of the coupling between these two parameters.

Second, the adaptation system may normalize its responses to sensory prediction errors with repeated exposure, akin to normalization processes observed in response to reward prediction errors^25^. For example, the system may increase its responses to small, yet persistent errors. Alternatively, responses to large errors may diminish over time until reaching some intermediate normalized update size. By this normalization hypothesis, the size of the motor correction changes over trials and, due to the invariance of the size of the clamped error, eventually converges on the same value for all error sizes.

To this point, we have assumed that the clamped visual error is the primary signal driving the change in behavior; however, other error signals, in particular signals arising from proprioception, may also impact adaptation to a visual perturbation. Thus, a third possibility is that the asymptotic response may reflect the limit of proprioceptive recalibration, which is independent of visual error size. That is, as the heading angle changes due to the clamped visual error, the proprioceptive sensory prediction error would increase, but with the opposite sign. The asymptote would correspond to the balance point between these two opposing error signals.

Future work will be required to formalize these hypotheses and develop experimental tests to evaluate the different mechanisms. Regardless of the appropriate reformulation of models of sensorimotor adaptation, we expect it will be fruitful to shift the focus away from the error sensitivity of the learning system, and instead, address the constraints on the behavioral change that arises in response to the error.

## Methods

### Participants

Healthy, young adults (*N* = 126, 89 females, age = 21 ± 2 years old) were recruited from the University of California, Berkeley, community. Each participant was tested in only one experiment. All participants were right-handed, as verified with the Edinburgh Handedness Inventory^26^. Participants received course credit or financial compensation for their participation. The Institutional Review Board at UC Berkeley approved all experimental procedures.

### Experimental apparatus

The participant was seated at a custom-made tabletop housing an LCD screen (53.2 cm by 30 cm, ASUS), mounted 27 cm above a digitizing tablet (49.3 cm by 32.7 cm, Intuos 4XL; Wacom, Vancouver, WA). The participant made reaching movements by sliding a modified air hockey “paddle” containing an embedded stylus. The position of the stylus was recorded by the tablet at 200 Hz. The experimental software was custom written in Matlab, using the Psychtoolbox extensions^27^.

### Reaching task

Center-out planar reaching movements were performed from the center of the workspace to targets positioned at a radial distance of 8 cm. Direct vision of the hand was occluded by the monitor, and the lights were extinguished in the room to minimize peripheral vision of the arm. The start location and target location were indicated by white and blue circles, respectively (both 6 mm in diameter).

To initiate each trial, the participant moved the digitizing stylus into the start location. The position of the stylus was indicated by a white feedback cursor (3.5 mm diameter). Once the start location was maintained for 500 ms, the target appeared at one of 8 locations, placed in 45° increments around a virtual circle. Participants were instructed to accurately and rapidly “slice” through the target, without needing to stop at the target location. Visual feedback, when presented, was provided during the reach until the movement amplitude exceeded 8 cm. As described below, the feedback either matched the position of the stylus (veridical) or followed a fixed path (clamped). If the movement was not completed within 300 ms, the words “too slow” were generated by the sound system of the computer.

In Experiment 1 (see below), the position of the cursor was frozen for 1 s once the movement amplitude reached 8 cm. The participant was free to begin moving back to the start location during this time. After the spatial feedback period, the cursor disappeared. Once the participant’s hand was back within 2 cm of the start circle, a white ring appeared, indicating the radial distance between the hand and center start position. The ring was displayed to aid the participant in returning to the start location, without providing angular information about hand position. Two changes were made in Experiment 2 (see below): First, the cursor was turned off 50 ms after the hand crossed the virtual target ring. Second, during the return movement, the feedback cursor reappeared when the participant’s hand was within 1 cm of the start. These changes reduced the time required for each trial and allowed the participants to complete the extended number of trials required in Experiment 2 within our time constraints. Average total trial time in Experiment 1 was 4.45 ± .62 s vs. 2.51 ± .26 s in Experiment 2.

### Experimental feedback conditions

Across the experimental session, there were three types of visual feedback. On no-feedback trials, the cursor disappeared when the participant’s hand left the start circle and only reappeared at the end of the return movement. On veridical feedback trials, the cursor matched the position of the stylus during the 8 cm outbound segment of the reach. On clamped feedback trials, the feedback followed a path that was fixed along a specific heading angle^17,28,29^. The radial distance of the cursor from the start location was still based on the radial extent of the participant’s hand during the 8 cm outbound segment, but the angular position was fixed relative to the target (i.e., independent of the angular position of the hand).

The primary instructions to the participant remained the same across the experimental session: Specifically, that they were to reach directly towards the visual target. Prior to the introduction of task-irrelevant clamped feedback trials, participants were briefed about the feedback manipulation. They were informed that the position of the cursor would now follow a fixed trajectory and that the angular position would be independent of their movement. They were explicitly instructed to ignore the cursor and continue to reach directly to the target. The same instructions in abbreviated form (“Ignore the cursor and move your hand directly to the target location”) were repeated verbally and with onscreen text after 20 movement cycles in Experiment 1 (exact mid-point) and every 40 movement cycles during Experiment 2.

### Experiment 1

In a previous experiment, adaptation to task-irrelevant clamped visual feedback was statistically uniform to offsets between 7.5°-95°. The main goal of Experiment 1 was to investigate if there was a dependency on error size for angles smaller than 7.5°. Participants (*n* = 96, 12 per group) were randomly assigned to one of eight groups that differed in terms of the size of the clamped visual feedback: 1°, 1.75°, 3.5°, 6°, 10°, 15°, and 45° (with a 0° group included as a control). The Euclidean distances for these clamp offsets, measured from the centers of cursor and target, were as follows (smallest to largest, in mm): 0, 1.4, 2.4, 4.9, 8.4, 13.9, 20.9, and 61.2. Given that the target diameter was 6 mm and the feedback cursor diameter was 3.5 mm, a substantial portion of the cursor overlapped with the target for the 1° and 1.75° clamps, and was fully embedded in the case of the 0° clamp. Half of the participants trained with a clockwise clamp offset, and the other half with a counterclockwise clamp offset.

The session began with two baseline blocks, the first comprised of 5 movement cycles (40 reaches to 8 targets) without visual feedback and the second comprised of 5 cycles with a veridical cursor displaying hand position. The experimenter then informed the participant that the visual feedback would no longer be veridical and would now be clamped at a fixed angle from the target location. The clamp block had 40 cycles. A short break (<30 s), as well as a reminder of the task instructions, was provided at the mid-way point of this block. Immediately following the perturbation block, there were two washout blocks, first a five cycle block in which there was no visual feedback, followed by five cycles with veridical visual feedback. Participants were debriefed at the end of the experiment and asked whether they ever intentionally tried to reach to locations other than the target. All subjects reported aiming to the target throughout the experiment.

### Experiment 2

In Experiment 2 we assessed adaptation over an extended number of task-irrelevant clamped visual feedback trials. The purpose of extending the perturbation block was to ensure that participants reached asymptotic levels of learning. We were particularly interested in whether asymptotic adaptation would converge in response to small and large clamps.

Participants (*n* = 30, 10 per group) were assigned to either a 1.75°, 3.5°, or a 15° clamped visual feedback group. Clockwise and counterclockwise perturbations were counterbalanced within each group. As in Experiment 1, the session started with two baseline blocks, five cycles without visual feedback and then 5 cycles with veridical feedback. However, the number of trials in the clamped visual feedback block was quadrupled to 160 cycles. We included one cycle with no visual feedback after every 40 movement cycles. The purpose of these interspersed no-feedback trials was to gauge adaptation magnitudes in the absence of the learning stimulus (i.e., clamped visual feedback) at different time points within the extended clamp block (Supplementary Fig. 3). Immediately prior to the no-feedback block, the participant was informed that there would be a few trials without feedback and reminded to always reach directly to the target. The experiment ended with a final block of five cycles with veridical visual feedback of the participant’s hand position.

### Comparison of error sensitivity and error correction

For the comparison of error sensitivity and error correction functions in Fig. 3, we used the early adaptation rate (panels a and b) and aftereffect data (panels c and d) from the present study, as well as data sets from three other studies that have compared adaptive responses to a range of error sizes^10,17,23^. The data from Experiments 1 and 2 in Wei and Kording^10^ were transformed from Cartesian coordinates (as presented in their paper) to polar coordinates. The data from Bond and Taylor^23^ were restricted to the aftereffect data from their Experiment 3 (comparison of adaptation to different rotation sizes). We used the data from the initial adaptation cycles (mean change in hand angle over first ten movement cycles) and aftereffect phase from Experiment 4 of Morehead et al.^17^ (comparison of different clamp offsets). To obtain measures of error sensitivity, the raw response magnitudes were divided by their corresponding error size.

### Data analysis

All statistical analyses and modeling were performed using Matlab 2015b and the Statistics Toolbox. The primary dependent variable in all experiments was endpoint hand angle, defined by the angle of the hand position relative to the target at the time the radial distance of the hand reached 8 cm from the start position (i.e., angle between lines connecting start position to target and start position to hand). Additional analyses were performed using hand angle at peak radial velocity rather than endpoint hand angle. The results were essentially identical for the two dependent variables; as such, we only report the results of the analyses using endpoint hand angle.

Outlier responses were removed from the analyses. To identify these, the Matlab “smooth” function was used to calculate a moving average (using a 5-trial window) of the hand angle data for each target location. Outliers were trials in which the observed hand angle deviated by >3 SD from the moving average function. This procedure resulted in the elimination of ~1% of trials involved in our statistical analyses of early adaptation rates and aftereffects; our findings are the same whether tests were performed with or without outlier removal (values reported in main text are with outlier removal). In total, less than 1% of trials overall, with a maximum of 2% for an individual, were removed.

Movement cycles consisted of eight consecutive reaches (one reach/target). Early adaptation rate was quantified by averaging the endpoint hand angle values over cycles 3–7 of the clamp, and dividing by the number of cycles (i.e., five) to get an estimate of the per-trial rate of change in hand angle. (As a check, we performed a secondary analysis using cycles 2–10 and obtained nearly identical results.) We opted to use this measure of early adaptation rather than obtain parameter estimates from exponential fits since the latter approach gives considerable weight to the asymptotic phase of performance and, therefore would be less sensitive to early differences in rate. This would be especially problematic in Experiment 2. The aftereffect was quantified by using the data from the first no-feedback cycle following the last clamp cycle. Details for all four no-feedback cycles in Experiment 2 are provided in the Supplemental section.

All t-tests were two-tailed. In order to confirm that there was a robust adaptive response in Experiment 1, a paired *t*-test was performed comparing baseline hand angle during the last cycle of the veridical feedback baseline to the first no-feedback cycle (i.e., aftereffect) immediately following the perturbation block. Post hoc tests following significant ANOVAs were performed using Tukey–Kramer’s Honest Significant Difference in order to determine specific differences in group means. Partial eta squared (*η*^2^) values are provided as a measure of effect size.

For the segmented linear regression (SLR) performed in Experiment 1, contiguous regression lines were fit to the data, with each line having an independent intercept and slope. Parameters for the regression lines were identified by a least-squares fitting procedure. Boundaries for the adjoining segments were estimated by finding the break point(s), an additional parameter defining where two separate regression lines meet, that minimized the residual sum of squares. Relative fits were compared using corrected Akaike Information Criterion (AICc) values, a procedure that adjusts for the number of data points and assigns penalties for extra parameters.

No statistical methods were used to predetermine sample sizes. The chosen sample sizes were based on our previous study using the clamp method^17^, as well as prior psychophysical studies of human sensorimotor learning^29–32^.

### Reaction and movement times

Average movement times were quite fast, averaging 119 ± 29 ms in Experiment 1 and 136 ± 25 ms in Experiment 2. The instructions did not impose any constraints on reaction time. On average, in Experiment 1 participants initiated their reaches in 429 ± 72 ms, while in Experiment 2 reaction times were 364 ± 54 ms. No significant correlations were found between these temporal variables and our primary measures of adaptation (rate and aftereffect magnitude).

### Data availability

All code and data are available upon request.

## Electronic supplementary material

Supplementary Information
