# Supplementary material for: Invariant errors reveal limitations in motor correction rather than constraints on error sensitivity
Source: Commun Biol. 2018 Mar 22;1:19. doi: 10.1038/s42003-018-0021-y (PMC6123629; doi:10.1038/s42003-018-0021-y)
Supplement: Supplementary file 1 — Supplementary Information [file 42003_2018_21_MOESM1_ESM.pdf]

SUPPLEMENTAL INFORMATION

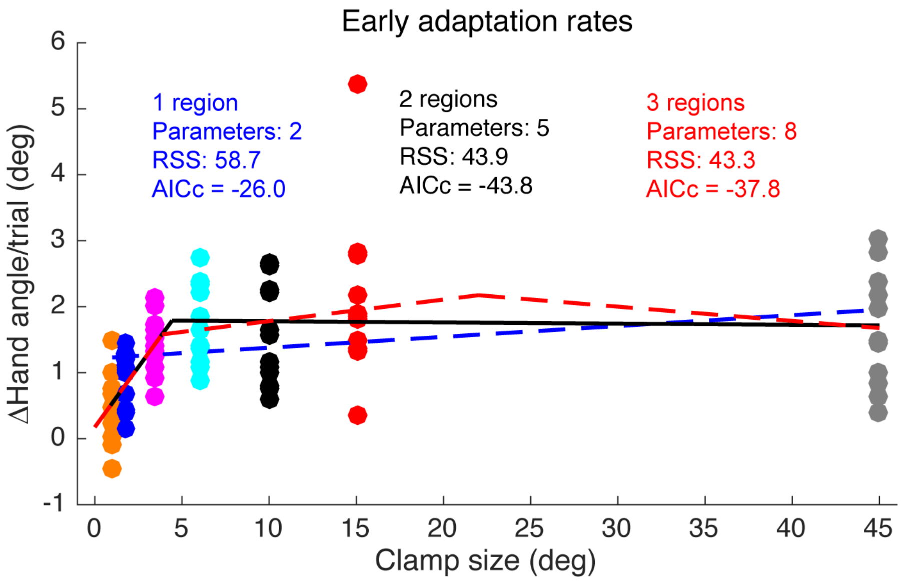

Supplementary Figure 1

Segmented regression analysis of early adaptation rates. In our previous study using the clamped feedback method, we observed similar adaptation rates for clamp offsets of 7.5° - 95°. In the current study, we focus on clamps < 7.5°. As described in the text, there was a clear scaling of adaptation rates for the smallest clamps (Figs. 1e, 2a). To determine the error size at which adaptation rates saturate (i.e., the breakpoint), we performed a segmented regression analysis, a widely used method for fitting data that are hypothesized to have at least two different linear regions[3]. This figure shows the results for a simple linear regression (1 region) and two segmented regression models, one with 2 regions and the other with 3 regions, along with the residual sum of squares (RSS) and corrected Akaike Information Criterion (AICc) scores. The best model, as determined by the AICc scores, was the two-region model. Models with more than three regions scored progressively worse and are not shown. Consistent with our prediction of a breakpoint < 7.5°, the predicted breakpoint was 4.4°.

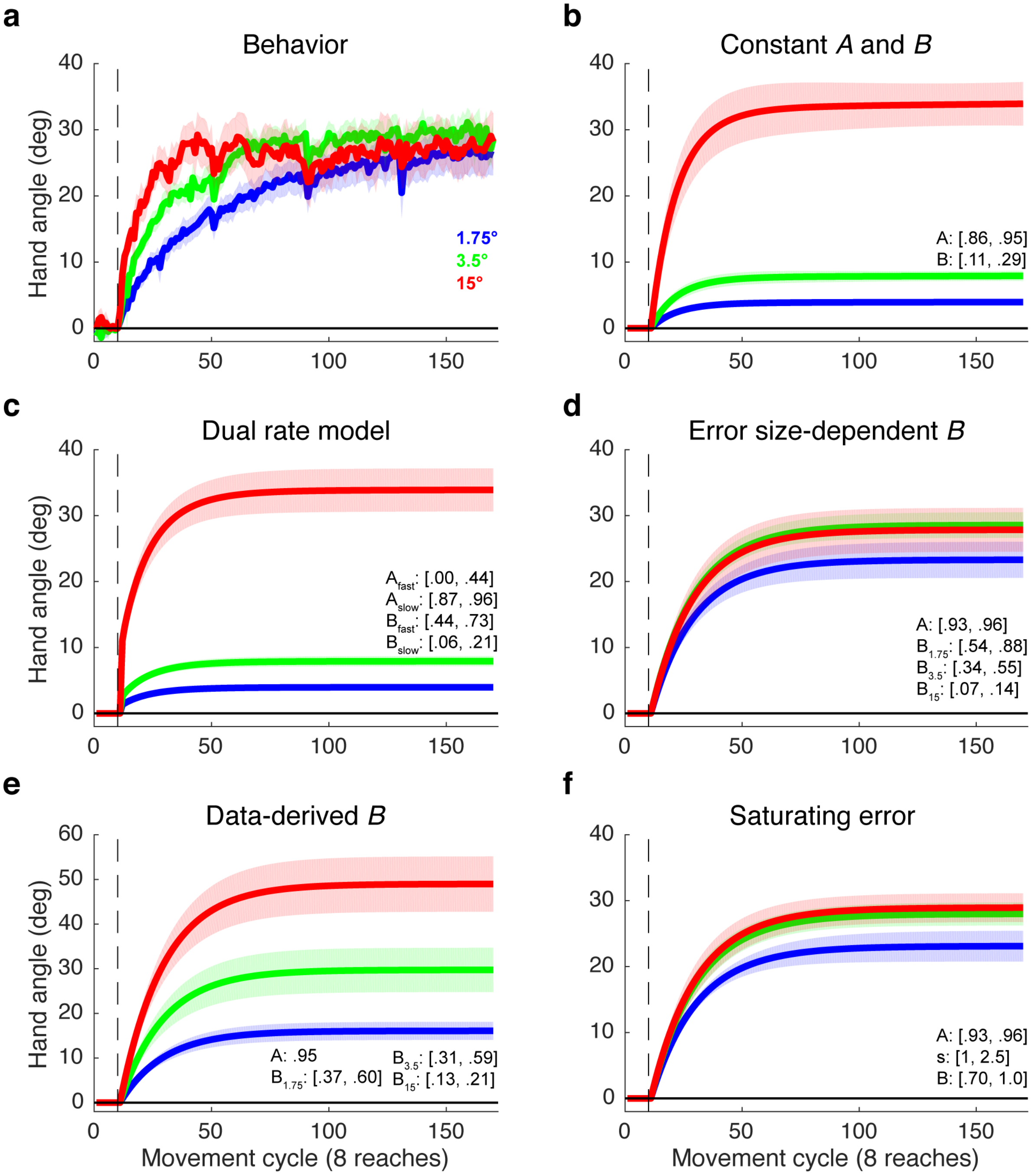

Supplementary Figure 2

Existing models fail to capture both early learning and asymptotic behavior observed during adaptation to different error sizes. (**a**) Behavioral data from Experiment 2 (same as Fig. 2 of main text, but with no-feedback cycles removed). (**b**) Simulation of a single rate state-space model with a common retention factor and learning rate (best fit *A* and *B* of bootstrapped group means). **(c)** Simulation of a dual-rate model, with separate retention and learning rate parameters that operate over different time scales (e.g., fast and slow). (**d**) Simulation using a common retention factor and error size-dependent learning rates

(best fit *A* and learning rates, B_e, for each clamp size). The simulated functions predict indistinguishable adaptation rates for the 3.5° and 15° clamp conditions over the initial portion of the perturbation block, in contrast to the markedly different rates observed in the actual behavior. **(e)** Simulation using estimates of *B* derived from the behavioral data. The functions approximate the early separation between learning functions, but predict divergent asymptotes. (Note: y-axis scaling was changed to fit data). **(f)** Based on a model from Tanaka et al. (2012), a saturating error function can capture the nonlinear effects of error size on adaptation. However, similar to the fits in ***d***, this model also predicts similar early adaptation rates for the three error sizes, as well as a lower asymptote for the 1.75° clamp. 95% CIs for parameter estimates in brackets. Lines and shading denote mean and SEM, respectively.

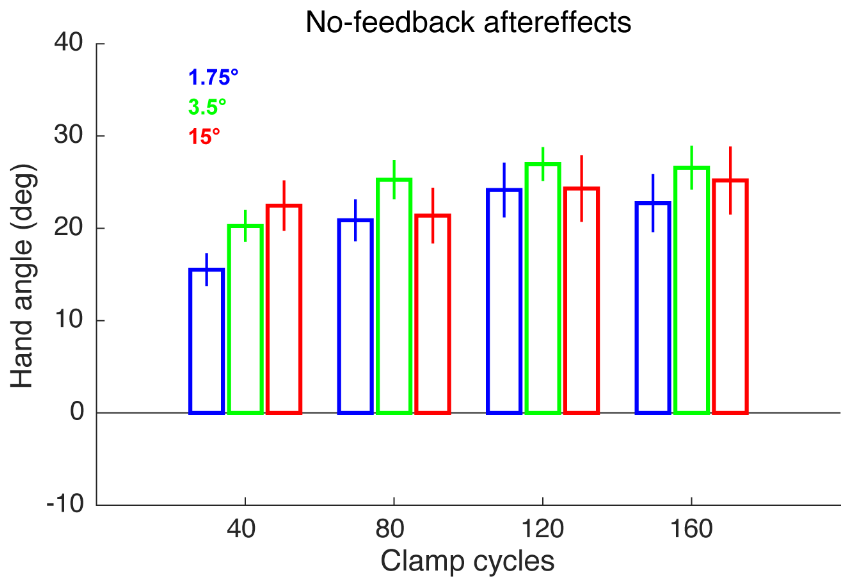

Supplementary Figure 3

Data for all of the no-feedback probes (x-axis represents number of clamp cycles preceding the no-feedback probe) from Experiment 2. The results of a two-way repeated measures ANOVA revealed no main effect for group (between subjects factor: $F_{2,27}$=.62, p=.54, η^2=.04), a significant effect for probe (within subjects factor: $F_{3.81}$=19.2, p<10^{-7}, η^2=.07), and a significant interaction effect ($F_{6,81}$=2.64, p=.02, η^2=.02). Due to the interaction, separate between subjects one-way ANOVAs were conducted for each of the four no feedback cycles. There was a marginal effect of group (i.e., clamp size) at the first probe only ($F_{2,27}$ = 2.77; p = .08; η^2=.21). Over subsequent blocks learning functions for each group began to converge, resulting in the corresponding values for no-feedback probes 2-4: $F_{2,27}$ = .92, .30, .39; p= .41, .75, .68; η^2= .07, .02, .03).

Supplementary Note 1.

Aftereffect data

In Experiment 1, there was an effect of error size on the aftereffect data obtained after 40 cycles of exposure to the clamped feedback ($F_{6,77}$=3.25, p=.007, η^2=.20, Fig. 1g). However, this effect was driven by the 1° group, and likely due to the trivial reason that the design did not include a sufficient number of training cycles for this group to reach asymptote. A Tukey-Kramer posthoc test revealed the only significant differences were between the 1° group and larger clamp sizes. Furthermore, excluding the 1° group, there were no reliable differences among all of the other pairwise comparisons (all t_{22}<2.07, all p>.05). Indeed, the magnitude of the aftereffect was not correlated with error size (r_{82}=0.04, p=0.71). These results suggested that asymptotic adaptation may be independent of error size, a hypothesis which was more rigorously tested in Experiment 2.

Supplementary Note 2.

Comparisons between Experiment 1 and Experiment 2

Overall, the magnitudes of final aftereffects in Experiment 2 were considerably larger than the aftereffect of matched groups in Experiment 1. One reason for this is, of course, the increase in the number of clamp cycles in Experiment 2, which had 160 compared to the 40 cycles used in Experiment 1. As can be seen in Supplementary Figure 3, the magnitude of adaptation is still rising after 40 cycles of clamped feedback. As an exploratory analysis, we performed a between-experiment 2-way ANOVA, with one factor being experiment (1 or 2) and the other error size, limiting this to the three conditions in Experiment 1 that were included in Experiment 2 (1.75°, 3.5° and 15°). We compared the aftereffect data after 40 clamp cycles (i.e., final aftereffects from Experiment 1 compared to first no feedback probe in Experiment 2). There was a significant effect of experiment ($F_{1,60}$=5.27, p=.03, η^2=.07), a marginal effect of clamp size ($F_{2,60}$ = 2.8, p=.07, η^2=.08), and no interaction ($F_{2,60}$=.57, p=.57, η^2=.02). These results suggest that some performance differences may have arisen from the methodological changes introduced in Experiment 2, namely, eliminating frozen endpoint feedback and utilizing a quicker method for finding the start position before each trial (see Methods). This resulted in a shorter total trial duration in Experiment 2, likely reducing the decay of learning associated with delay[16]. This point highlights that asymptotic magnitude is context specific, as adaptation is highly sensitive to variables such as temporal delay between hand and cursor movement[17,18], viewing angle[19], and the nature of visual feedback (e.g., online versus endpoint feedback)[20,21]. As such, our claim that the asymptote is independent of error size holds for a given context; the value will shift in other contexts, although the shift will be uniform for all error sizes.

Supplementary Note 3.

Computational models of sensorimotor adaptation predict divergence of asymptotic adaptation when early adaptation rates are different.

A single rate state-space model can generally provide a reasonable account of the performance changes observed in standard adaptation studies in which the feedback is contingent on the movement[4–6]. This model takes the following form:

$$z_{n+1} = Az_n + Be_n \qquad (1)$$

$$\hat{y}_n = -z_n \qquad (2)$$

z_n represents the state estimate of the perturbation on trial n. The learning rate, B, corresponds to the proportion of the error, e_n, that is corrected for, and A is a retention factor which represents the proportion of the state retained from one trial to the next. The reach direction relative to the target on trial n, $\hat{y}_{n,}$, is opposite in sign to the state estimate. Note that unlike standard adaptation experiments in which the error changes over trials, the error remains constant with the clamp method.

We evaluated different state-space models, focusing on the data from Experiment 2 (reproduced in Supplementary Fig. 2a). For our model fitting and simulation procedures we applied standard bootstrapping techniques, constructing group-averaged hand angle data 1000 times by randomly resampling with replacement from the participant pool. Using Matlab's *fmincon* function, we estimated the retention and learning parameters which minimized the least squared error between the bootstrapped data and model output ($\hat{y}_n$). All values in brackets represent the 95% bootstrapped C.I. of the resampled means.

We first verified that a single rate state-space model, which in its basic form assumes single, constant A and B values for all error sizes, cannot account for the key features of the current experiments. We

estimated A and B for each of the 1000 bootstrap simulations ([.86, .95] and [.11, .29] for A and B, respectively) (Supplementary Fig. 2b). In contrast to the invariant asymptotes observed in Experiment 2, the simulated functions generated with these parameter estimates showed wildly different asymptotic values: [3.2°, 4.8°], [6.5°, 9.6°], and [27.9°, 41.0°] in response to 1.75°, 3.5°, and 15° clamps, respectively. As the error term is constant during a clamp (i.e., e_n is equal to clamp size on every trial), and the asymptote is equal to $(Be)/(1-A)$, different error terms (i.e., clamp sizes) will always generate different asymptotic values if A and B are both fixed.

An alternative class of models posit that performance changes reflect the activity of multiple learning processes, each operating in a similar manner but over different time scales[7,8]. For example, in a dual-rate state-space model of adaptation[8], the output (5) is the sum of two single rate models, one in which both learning and forgetting occur over a faster time scale (3) than the other (4). Despite this added flexibility, this model is similarly constrained as the single rate model. As previously explained, the asymptotes for each state will still be equal to $(Be)/(1-A)$, meaning different size clamps eventually reach different asymptotes. Indeed, our simulations show that a dual rate state-space model will generate functions (Supplementary Fig. 2c) which are qualitatively similar to the single rate model (Supplementary Fig. 2b).

$$z_f(n+1) = A_f z_f(n) + B_f e(n) \qquad (3)$$

$$z_s(n+1) = A_s z_s(n) + B_s e(n) \qquad (4)$$

$$\text{where } A_f < A_s,\ B_f > B_s$$

$$z = z_f + z_s \qquad (5)$$

As noted in the main text, various studies have suggested that the learning rate, B, may vary with error size[9–14]. To examine this class of models, we used a single rate state-space model (eqns. 1 and 2) with a common retention factor and three learning rates, one for each error (clamp) size. We again obtained

1000 sets of parameter estimates from the bootstrapped data in order to generate the simulations seen in Supplementary Fig. 2d. This 4-parameter model (a common *A*, and separate *B* values for each clamp size) also failed to capture the key features of the behavior. Although the model generated more similar asymptotes across clamp sizes than the single rate state-space model with constant *A* and *B* ([18.4°, 28.8°], [25.1°, 32.6°] and [21.6°, 34.5°] for 1.75°, 3.5°, and 15° clamps), it failed to capture the clear separation of early adaptation rates observed in the behavioral data (Supplementary Fig. 2a). In fact, the model predicted nearly identical early adaptation rates (i.e., mean change in hand angle per movement cycle over first 5 cycles) for the 3.5° and 15° clamps ([.9°, 1.4°] and [.7°, 1.5°]), and only a modestly slower rate for the 1.75° clamp [.7°, 1.1°]. Moreover, *B* values in the adaptation literature[4,5,15] typically range from .10 to .30, whereas the *B* values required to fit the data for the small error clamps would have to go as high as .70, and even then, provide a relatively poor match to the observed behavior.

We also considered an alternative means of parameter estimation with this model (eqns. 1 and 2) in which we recapitulate the different adaptation rates by using the best fit *A* value of .95 and approximating the mean *B* values directly from the early cycles of the bootstrapped data, using the mean change in hand angle over the first 5 cycles divided by clamp size. This procedure yielded the following *B* values: [.36, .60], [.31, .59], and [.13, .21] for 1.75°, 3.5°, and 15° clamps, respectively. Here the predicted asymptotes in response to the three clamp sizes again strayed far from the observed behavior: [12.2°, 20.1°], [20.4°, 39.3°], and [36.4°, 60.4°].

Lastly, we tested a model[13] motivated by observations suggesting that the perceived error is linear for small errors before saturating at an upper bound for larger errors[9]. To model this nonlinear effect, the authors replaced the error term, *e*, with a function which saturates for larger errors ($s*tanh(e_n/s)$). *s* is a scaling parameter for the hyperbolic tangent function:

$$z_{n+1} = Az_n + B*s*tanh(e_n/s)$$

This model produced learning functions which suffered from the same shortcomings as the state-space model with error-dependent learning rates, again predicting virtually identical early adaptation rates for the 3.5° and 15° clamp ([.8°, 1.3°] and [.8°, 1.5°]), a *B* value [.70, 1.0] which far exceeds normal estimates of this value, and a slightly lower asymptote for the 1.75° clamp ([18.7°, 27.4°] versus [24.8°, 31.6°] and [25.2°, 33.7°] for the 3.5° and 15° clamps, respectively).

In summary, even models that account for variation in sensitivity to error fail to capture the combined effects of divergent early adaptation rates and invariant asymptotic performance (see Moazzezi 2018 for a comprehensive analysis of this issue)[22].

Supplementary References

1. Vaswani, P. A. *et al.* Persistent Residual Errors in Motor Adaptation Tasks: Reversion to Baseline and Exploratory Escape. *J. Neurosci.* **35,** 6969–6977 (2015).
2. Scheidt, R. A., Conditt, M. A., Secco, E. L. & Mussa-Ivaldi, F. A. Interaction of Visual and Proprioceptive Feedback During Adaptation of Human Reaching Movements. *J. Neurophysiol.* **93,** 3200–3213 (2005).
3. Hudson, D. J. Fitting Segmented Curves Whose Join Points Have to Be Estimated. *J. Am. Stat. Assoc.* **61,** 1097–1129 (1966).
4. Galea, J. M., Mallia, E., Rothwell, J. & Diedrichsen, J. The dissociable effects of punishment and reward on motor learning. *Nat. Neurosci.* **18,** 597–602 (2015).
5. Huang, V. S., Haith, A., Mazzoni, P. & Krakauer, J. W. Rethinking motor learning and savings in adaptation paradigms: model-free memory for successful actions combines with internal models. *Neuron* **70,** 787–801 (2011).
6. Thoroughman, K. A. & Shadmehr, R. Learning of action through adaptive combination of motor primitives. *Nature* **407,** 742–747 (2000).
7. Kording, K. P., Tenenbaum, J. B. & Shadmehr, R. The dynamics of memory as a consequence of optimal adaptation to a changing body. *Nat. Neurosci.* **10,** 779–786 (2007).
8. Smith, M. A., Ghazizadeh, A. & Shadmehr, R. Interacting Adaptive Processes with Different Timescales Underlie Short-Term Motor Learning. *PLOS Biol.* **4,** e179 (2006).
9. Fine, M. S. & Thoroughman, K. Motor Adaptation to Single Force Pulses: Sensitive to Direction but Insensitive to Within-Movement Pulse Placement and Magnitude. *J. Neurophysiol.* **96,** 710–720 (2006).
10. Kasuga, S., Hirashima, M. & Nozaki, D. Simultaneous Processing of Information on Multiple Errors in Visuomotor Learning. *PLoS ONE* **8,** (2013).
11. Körding, K. P. & Wolpert, D. M. The loss function of sensorimotor learning. *Proc. Natl. Acad. Sci. U. S. A.* **101,** 9839–9842 (2004).

12. Marko, M. K., Haith, A. M., Harran, M. D. & Shadmehr, R. Sensitivity to prediction error in reach adaptation. *J. Neurophysiol.* **108,** 1752–1763 (2012).

13. Tanaka, H., Krakauer, J. W. & Sejnowski, T. J. Generalization and Multirate Models of Motor Adaptation. *Neural Comput.* **24,** 939–966 (2012).

14. Wei, K. & Körding, K. Relevance of Error: What Drives Motor Adaptation? *J. Neurophysiol.* **101,** 655–664 (2009).

15. Thoroughman, K. A. & Shadmehr, R. Learning of action through adaptive combination of motor primitives. *Nature* **407,** 742–7 (2000).

16. Kim, S., Oh, Y. & Schweighofer, N. Between-Trial Forgetting Due to Interference and Time in Motor Adaptation. *PLoS ONE* **10,** (2015).

17. Held, R., Efstathiou, A. & Greene, M. Adaptation to displaced and delayed visual feedback from the hand. *J. Exp. Psychol.* **72,** 887 (1966).

18. Kitazawa, S., Kohno, T. & Uka, T. Effects of delayed visual information on the rate and amount of prism adaptation in the human. *J. Neurosci.* **15,** 7644–7652 (1995).

19. Jalali, R., Miall, R. C. & Galea, J. M. No consistent effect of cerebellar transcranial direct current stimulation (tDCS) on visuomotor adaptation. *J. Neurophysiol.* jn.00896.2016 (2017). doi:10.1152/jn.00896.2016

20. Taylor, J. A. & Ivry, R. B. Flexible Cognitive Strategies during Motor Learning. *PLoS Comput. Biol.* **7,** e1001096 (2011).

21. Taylor, J. A., Krakauer, J. W. & Ivry, R. B. Explicit and implicit contributions to learning in a sensorimotor adaptation task. *J Neurosci* **34,** 3023–3032 (2014).

22. Moazzezi, R. A new model for Cerebellar computation. Preprint at https://arxiv.org/abs/1802.08217 (2018).
